# Nef functions in BLT mice to enhance HIV-1 replication and deplete CD4^+^CD8^+^ thymocytes

**DOI:** 10.1186/1742-4690-9-44

**Published:** 2012-05-28

**Authors:** Wei Zou, Paul W Denton, Richard L Watkins, John F Krisko, Tomonori Nochi, John L Foster, J Victor Garcia

**Affiliations:** 1 Division of Infectious Diseases, Center for AIDS Research, University of North Carolina, Chapel Hill, NC, 27599-7042, USA; 2 Division of Infectious Diseases, UNC Center for AIDS Research, 2042 Genetic Medicine, Campus Box 7042, Chapel Hill, NC, 27599-7042, USA

**Keywords:** HIV-1, Nef, Humanized mouse, Replication, Pathogenicity

## Abstract

**Background:**

The outcome of untreated HIV-1 infection is progression to AIDS and death in nearly all cases. Some important exceptions are the small number of patients infected with HIV-1 deleted for the accessory gene, *nef*. With these infections, disease progression is entirely suppressed or greatly delayed. Whether Nef is critical for high levels of replication or is directly cytotoxic remains controversial. The major problem in determining the role of Nef in HIV/AIDS has been the lack of tractable *in vivo* models where Nef’s complex pathogenic phenotype can be recapitulated.

**Results:**

Intravenous inoculation (3000 to 600,000 TCIU) of BLT humanized mice with HIV-1_LAI_ reproducibly establishes a systemic infection. HIV-1_LAI_ (LAI) replicates to high levels (peak viral load in blood 8,200,000 ± 1,800,000 copies of viral RNA/ml, range 3,600,000 to 20,400,000; n = 9) and exhaustively depletes CD4^+^ T cells in blood and tissues. CD4^+^CD8^+^ thymocytes were also efficiently depleted but CD4^+^CD8^-^ thymocytes were partially resistant to cell killing by LAI. Infection with a *nef*-deleted LAI (LAINef*dd*) gave lower peak viral loads (1,220,000 ± 330,000, range 27,000 to 4,240,000; n = 17). For fourteen of seventeen LAINef*dd*-infected mice, there was little to no loss of either CD4^+^ T cells or thymocytes. Both LAI- and LAINef*dd*-infected mice had about 8% of total peripheral blood CD8^+^ T cells that were CD38^+^HLA-DR^+^ compared <1% for uninfected mice. Three exceptional LAINef*dd*-infected mice that lost CD4^+^ T cells received 600,000 TCIU. All three exhibited peak viral loads over 3,000,000 copies of LAINef*dd* RNA/ml. Over an extended time course, substantial systemic CD4^+^ T cell loss was observed for the three mice, but there was no loss of CD4^+^CD8^+^ or CD4^+^CD8^-^ thymocytes.

**Conclusion:**

We conclude Nef is necessary for elevated viral replication and as a result indirectly contributes to CD4^+^ T cell killing. Further, Nef was not necessary for the activation of peripheral blood CD8^+^ T cells following infection. However, CD4^+^CD8^+^ thymocyte killing was dependent on Nef even in cases of elevated LAINef*dd* replication and T cell loss. This depletion of thymic T cell precursors may be a significant factor in the elevated pathogenicity of CXCR4 trophic HIV-1.

## Background

HIV-1 infection leads to the near total loss of CD4^+^ T cells and results in immune incompetence [1,2]. Nef is considered to be a critical inducer of pathogenicity for HIV-1, because there are several reported cases of human infection by HIV-1 lacking a functional *nef* that failed to develop AIDS for twelve years or more [3-9]. Also, support for an important role for simian immunodeficiency virus Nef in pathogenesis and disease progression comes from elegant experiments performed in non-human primates where the absence of Nef resulted in delayed disease progression [10,11].

In *vivo* and *ex vivo* models of HIV-1 infection have been utilized to assess the role of Nef in viral replication and pathogenesis. Transgenic mouse models have demonstrated that Nef is the only HIV-1 protein that has direct pathogenic effects in mice [12-14]. Results from an HIV-1 infection model employing *ex vivo* cultures of human tonsil suggested a role for Nef as a replication factor [15-18]. *Ex vivo* experiments with human fetal thymus organ culture (HF-TOC) found that *nef* functioned as a pathogenic factor that does not enhance replication [19]. The findings with HF-TOC were confirmed with the SCID-hu thy/liv implant model in which infection can be extended beyond the maximum two-week duration for most *ex vivo* models [19]. Other groups found a dual role for Nef in HIV-1 infection of SCID-hu thy/liv implants as a replication and a pathogenesis factor [20,21]. Aldrovandi *et al.* found the impact of *nef*-deleted HIV-1 on viral replication to be dependent on the initial dose [22,23].

To address Nef’s role in HIV-1 pathogenesis during prolonged systemic infection, we have investigated the impact of inactivating *nef* on HIV-1 infection *in vivo* using BLT humanized mice [24-27]. This advanced model for human immune system reconstitution combines human stem cell engraftment in bone marrow with a human fetal liver/thymus implant producing a full range of systemically disseminated human immune cells including B cells, monocytic cells, dendritic cells and T cells. Human thymocyte education occurs within the implanted thymus which is a fully human cellular compartment [24,26,28]. BLT humanized mice have both human T cells and human thymocytes that can be infected simultaneously. This distinction establishes the BLT mouse model as a novel system for determining *in vivo* pathogenesis attributable to HIV-1 accessory genes. We inoculated mice with the strictly CXCR4-tropic virus, HIV-1_LAI_ (LAI), to maximize the pathogenic impact of the infection [29,30]. At three different inoculums in BLT mice, LAI rapidly depletes human CD4^+^ T cells in the peripheral blood and in tissues and eliminates CD4^+^CD8^+^ thymocytes from the implanted human thymic tissue [27].

At a low intravenous inoculum, we found *nef*-defective LAI to be greatly delayed for replication with lower peak viral loads relative to wild-type LAI. There was minimal CD4^+^ T cell and CD4^+^CD8^+^ thymocyte killing. At a high dose, the delay in *nef*-defective LAI replication was largely lost and in a few exceptional cases there was gradual loss of CD4^+^ T cells in blood and tissues. However, in no case did *nef*-defective LAI infection result in loss of thymocytes.

With these findings we have established the humanized BLT mouse model as highly appropriate for directly studying the complex role of Nef in processes that are critical for disease progression during HIV-1 infection. Our results suggest an indirect role for Nef in T cell killing and a direct role for Nef in loss of thymic function in HIV-1 infection [19,31,32].

## Results

### Defining the course of infection in the BLT humanized mouse with the highly pathogenic HIV-1_LAI_

**Figure 1:**
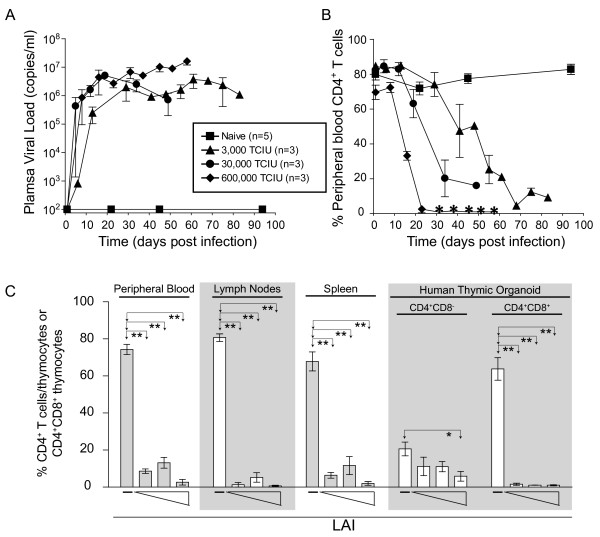
**BLT humanized mice exhibited a profound loss of human CD4**^**+**^**T cells/thymocytes following HIV-1**_**LAI**_**infection. (A-B)** Three cohorts of BLT humanized mice were exposed intravenously to increasing doses of HIV-1_LAI_ (LAI). LAI inoculations included a low dose of 3000 TCIU (triangles), an intermediate dose of 30,000 TCIU (circles), and a high dose of 600,000 TCIU (diamonds). A fourth cohort consisted of naïve BLT humanized mice that were not exposed to virus (squares). Longitudinal analysis of plasma viral load revealed efficient viral replication in each of the three groups of LAI infected mice regardless of the inoculation dose, and no virus replication was observed in the naïve animals (A). In the absence of infection in naïve BLT humanized mice, the percentage of human CD4^+^ T cells in the peripheral blood remains constant. In contrast, LAI infected mice showed an inoculation dose dependent difference in the kinetics of CD4^+^ T cell loss. Asterisks represent the absence of detectable levels of peripheral blood human CD4^+^ T cells in the BLT humanized mice infected with 600,000 TCIU of LAI at the time point indicated (B). **(C)** Regardless of the inoculation dose, systemic loss of CD4^+^ T cells was observed in infected BLT humanized mice when compared to naïve animals. Shown are the percentages of human CD4^+^ T cells present in peripheral blood, lymph nodes, and spleen, as well as the percentages of CD4^+^CD8^-^ and CD4^+^CD8^+^ thymocytes in the human thymic organoid. The percent of CD4^+^ T cells in peripheral blood or tissues was relative to total CD3^+^ T cells while the percent of CD4^+^CD8^-^ and CD4^+^CD8^+^ thymocytes was relative to total thymocytes. “—” indicates naïve mice (n = 5 in PB, spleen and HTO or n = 4 in LN). Triangles indicate the increasing doses: 3000 TCIU (n = 3), 30,000 TCIU (n = 3), 600,000 TCIU (n = 3). Unpaired two-tailed t tests were performed to compare the naïve mice to the mice in each LAI infection dose group within the same tissue. If no difference was detected, the comparison is unmarked (alpha = 0.05). Comparisons yielding significant differences are represented by a line connecting the arrows above the respective bars (*p < 0.05; **p < 0.01).

The goal of these experiments was to investigate the role of Nef in the pathogenic sequela of HIV-1_LAI_ (LAI) *in vivo*. For this purpose we used the BLT humanized mouse model that has been shown to recapitulate key aspects of HIV infection in humans [27,33,34]. We first investigated the relationship between the dose of viral inoculum and the course/outcome of LAI infection. The intravenous doses chosen were 3000 TCIU, 30,000 TCIU and 600,000 TCIU which correspond to 0.23 ng, 2.3 ng and 45 ng of p24^gag^. We designated 3000 TCIU as a low dose compared to inoculums of 12.5 and 25 ng of p24^gag^ directly injected into the thy/liv implant of SCID-hu mice in previously published studies [22,23]. When BLT mice were infected intravenously with a low dose of LAI (3000 TCIU), all mice became systemically infected. The virus replicated to a high level by two weeks and ultimately exceeded 10^6^ copies of viral RNA/ml in peripheral blood (Figure 1A). After the viral load reached about 10^6^ copies of HIV RNA/ml, a decrease in peripheral blood CD4^+^ T cells ensued (Figure 1B). CD4^+^ T cell depletion proceeded such that CD4^+^ T cell levels were very low by ten weeks. By contrast, naïve control mice had stable CD4^+^ T cell levels in the peripheral blood for the entire period of the experiment (Figure 1B). Increasing the infectious dose of virus by 10-fold (30,000 TCIU) gave an acceleration of viral production with 10^6^ copies of RNA/ml appearing in blood by two weeks (Figure 1A). Concomitant with the earlier appearance of viral RNA, there was a more rapid decline of CD4^+^ T cells (Figure 1B). Finally, a further 20-fold increase in viral inoculum (6X10^5^ TCIU) gave massive viral replication, yielding 10^7^ copies of viral RNA/ml of blood (Figure 1A). There was also a drastic loss of CD4^+^ T cells (Figure 1B) by 3 weeks. These results demonstrate that intravenous infection of BLT mice with LAI, at even the low dose, results in high levels of virus in blood and severe depletion of peripheral blood CD4^+^ T cells. The observed CD4^+^ T cell depletion is consistent with the pathogenic nature of CXCR4-trophic HIV infection in humans.

After establishing the effect of LAI infection on peripheral blood CD4^+^ T cell levels, we evaluated its effect on CD4^+^ cell depletion in lymph nodes, spleen and in the human thymic organoid (HTO). LAI depleted CD4^+^ T cells from lymph node and spleen at all doses (Figure 1C). The CD4^+^CD8^+^ double positive thymocytes were also efficiently depleted (Figure 1C). Interestingly, we noted that CD4^+^CD8^-^ single positive cells in the thymus were relatively resistant to depletion with only the highest inoculum significantly decreasing the level of these cells (Figure 1C). After evaluating the dose–response relationship of LAI and establishing its pathogenic potential in BLT mice, we next investigated the role of Nef.

### *Nef*-defective LAI, LAINef*dd*

**Figure 2:**
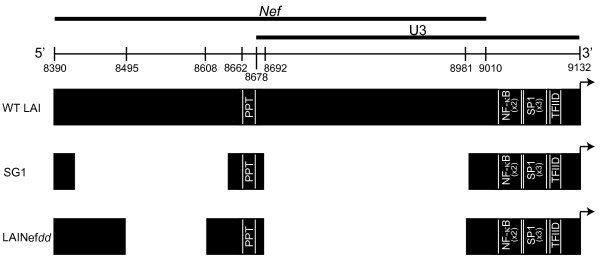
**Schematic of WT LAI and LAINef*dd*.** Three bars represent *nef* coding sequence and the U3 sequence for LAI, LAINef*dd* and patient SG1 clone 27 sequence [35]. SG1 clone 27 reflects the tendency of patients with defective *nef*s to lose all remaining coding sequence [5,7,35,36]. The coding sequence of WT type LAI *nef* (NCBI Accession, K02013) is presented in the top bar (nucleotides 8390–9010). PPT, polypurine tract is nucleotides 8662–8678. U3 consists of nucleotides 8678 to 9132. The core U3 promoter elements include two NF-κB binding sites, three SP1 sites and the TFIID binding site or TATA box. In the lower bar the 5’ deletion incorporated into LAINef*dd* is 114 nucleotides (8495–8608). Within the deletion, a four base insertion was added to create a frame shift (See Methods). The 3′ gap in LAINef*dd* is a frame-shifting deletion of 290 bases (8692–8981). In the middle bar the deletions in SG1 isolate 27 are aligned with LAI. The gaps are not numbered for simplicity of presentation. The 5′ deletion in SG1 is 217 nucleotides and the 3′ deletion is 292 nucleotides.

To follow the course of infection by LAI with an irreversibly inactivated *nef* (LAINef*dd*) in humanized BLT mice LAINef*dd* was constructed (Figure 2)*.* Two large deletions flanking the polypurine tract were introduced into *nef*. The double deletions reflect the long term convergent evolution of *nef*-defective virus in patients to lose all *nef* coding sequence except the PPT [5,7,35,36]. LAINef*dd* also models *nef*-defective virus from patients by maintaining conserved promoter elements in the 3′ end of U3 (Additional file 1: Figure S1). By a single round infection assay with TZM-bl cells, LAINef*dd* exhibited the expected reduction of virion infectivity [37,38]. The infectivity of LAINef*dd* was not significantly different from that of LAINefXhoI with a non-deleted, frame-shifted *nef* (LAINefXhoI, see Additional file 2: Figure S2).

### Infection of BLT mice with a low dose of LAINef*dd*

**Figure 3:**
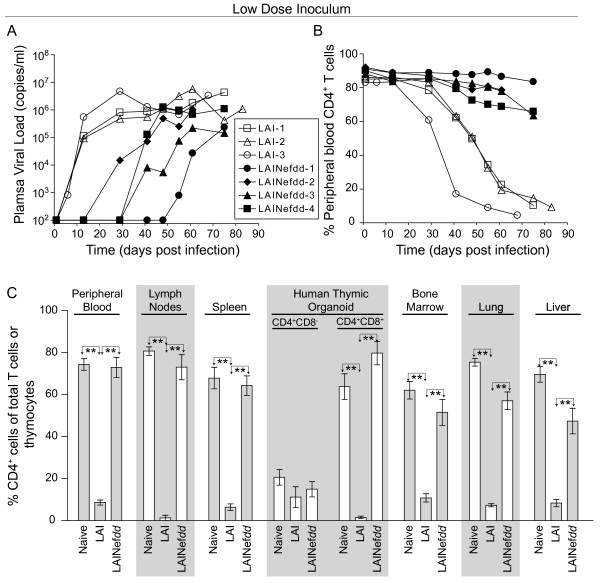
**Analysis of BLT humanized mice inoculated with a low dose of*nef*(−) or wild-type LAI. (A)** Each line depicts longitudinal plasma viral load data from individual BLT humanized mice infected with 3000 TCIU of LAINef*dd* (closed symbols) or LAI (open symbols). These data demonstrate delayed replication of LAINef*dd* relative to LAI following low dose inoculation. **(B)** Each line depicts the percentage of CD4^+^ T cells in peripheral blood over time where each animal’s symbol is matched to the mice in (A). Mice infected with a low dose of LAINef*dd* showed minimal changes in CD4^+^ T cell percentages when compared to BLT humanized mice inoculated with an equal dose of LAI. **(C)** Naïve BLT humanized mice (n = 5 in PB, spleen and HTO or n = 4 in LN, BM, lung and liver) and BLT humanized mice inoculated with 3000 TCIU of LAINef*dd* (n = 4) exhibited similar levels of CD4^+^ cells while mice inoculated with the same dose of LAI (n = 3) exhibited a drastic reduction in these cells. Shown are the percentages of human CD4^+^ T cells present in peripheral blood, lymph nodes, spleen, bone marrow, lung and liver, as well as the percentages of CD4^+^CD8^-^ and CD4^+^CD8^+^ thymocytes in the human thymic organoid. The percent of CD4^+^ T cells in peripheral blood or tissues was relative to total CD3^+^ T cells while the percent of CD4^+^CD8^-^ and CD4^+^CD8^+^ thymocytes was relative to total thymocytes. One-way ANOVA with three Bonferroni multiple comparisons tests was performed to compare the results within each tissue. If no difference was detected, the comparison is unmarked (alpha = 0.05). Comparisons yielding significant differences are represented by a line connecting the arrows above the respective bars (**p < 0.01).

Four mice inoculated with 3000 TCIU of LAINef*dd* (0.56 ng p24^gag^) became systemically infected. The appearance of virus in peripheral blood was greatly delayed from the 7–14 days seen for wild-type LAI-infected mice (Figure 3A, *LAI-1,-2* and −*3*) to 30–55 days for the four LAINef*dd* infected mice (*p* = 0.014, log rank test). These results demonstrate a generally reduced level of replication by the *nef*-deleted virus. However, in two of four mice (LAINef*dd*-2 and LAINef*dd*-4) viral loads did reach 10^6^ copies of viral RNA/ml of plasma demonstrating the *in vivo* fitness of *nef*-defective virus (Figure 3A). The other two mice (LAINef*dd*-1 and LAINef*dd*-3) had depressed viral replication with viral loads clearly under 10^6^ after sixty days. In contrast to the mice infected with wild-type LAI, the LAINef*dd* infected mice did not show significant depletion of their circulating CD4^+^ T cells (Figure 3B). Even the two mice whose viral loads reached 10^6^ copies/ml maintained high levels of CD4^+^ T cells in peripheral blood (Figure 3B). Therefore, infection with a low dose of *nef*-deleted virus results in delayed replication and most strikingly a minimal capacity to induce peripheral CD4^+^ T cell depletion.

To further explore the impact of *nef*-defective HIV infection, CD4^+^ T cells in lymphocyte containing tissues were analyzed. As seen in Figure 3C, LAINef*dd* infection did not result in depletion of CD4^+^ T cells in lymph node (LN), spleen, bone marrow (BM), lung and liver reflecting what was observed in peripheral blood (Figure 3B and C). Consistent with the fact that at this low dose wild-type LAI did not induce a severe depletion of CD4^+^CD8^-^ thymocytes, infection with the LAINef*dd* also did not result in CD4^+^CD8^-^ thymocyte depletion (Compare Figure 1C and 3C). However, a dramatic difference between LAI and LAINef*dd* infections was noted in the levels of CD4^+^CD8^+^ thymocytes present in the implanted human thymus. Specifically, infection with wild-type virus resulted in a severe depletion of double positive thymocytes, but infection with the *nef*-deleted virus did not deplete these cells (Figure 3C). These data indicate that 3000 TCIU of LAINef*dd* is sufficient to establish an infection in BLT humanized mice. However, consistent with Nef’s role as an important replication and pathogenic factor LAINef*dd* was greatly reduced in its ability to initiate replication and to induce systemic CD4^+^ T cell and double-positive thymocyte depletion.

### Infection of BLT mice with an intermediate dose of LAINef*dd*

**Figure 4:**
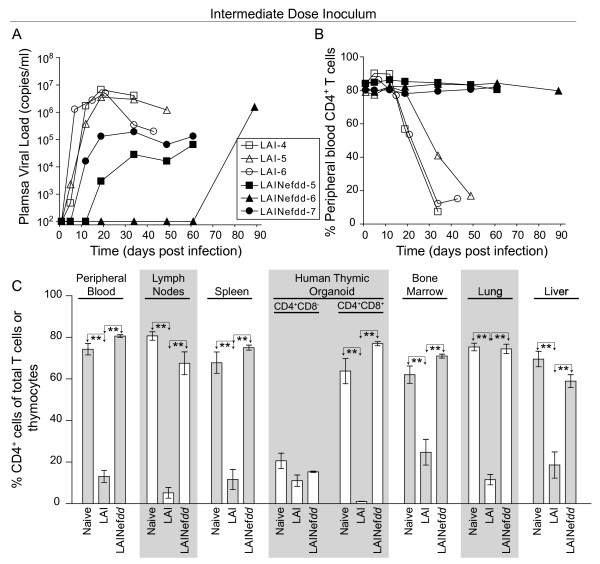
**Analysis of BLT humanized mice inoculated with an intermediate dose of*nef*(−) or wild-type LAI. (A)** Each line depicts longitudinal plasma viral load data from individual BLT humanized mice infected with 30,000 TCIU of LAINef*dd* (closed symbols) or LAI (open symbols). Two LAINef*dd* infected mice and all three LAI infected mice showed detectable viremia by three weeks post-exposure while one LAINef*dd* infected mouse exhibited delayed detection of viremia. **(B)** Each line depicts the percentage of CD4^+^ T cells in peripheral blood over time where each animal’s symbol is matched to the mice in (A). Mice infected with an intermediate dose of LAINef*dd* showed no changes in CD4^+^ T cell percentages whereas BLT humanized mice inoculated with an equal dose of LAI exhibited a decline of these cells. **(C)** Naïve BLT humanized mice (n = 5 in PB, spleen and HTO or n = 4 in LN, BM, lung and liver) and BLT humanized mice inoculated with 30,000 TCIU of LAINef*dd* (n = 3) exhibited similar levels of CD4^+^ cells while mice inoculated with the same dose of LAI (n = 3) exhibited a drastic reduction in these cells. Shown are the percentages of human CD4^+^ T cells present in peripheral blood, lymph nodes, spleen, bone marrow, lung and liver, as well as the percentages of CD4^+^CD8^-^ and CD4^+^CD8^+^ thymocytes in the human thymic organoid. The percent of CD4^+^ T cells in peripheral blood or tissues was relative to total CD3^+^ T cells while the percent of CD4^+^CD8^-^ and CD4^+^CD8^+^ thymocytes was relative to total thymocytes. One-way ANOVA with three Bonferroni multiple comparisons tests was performed to compare the results within each tissue. If no difference was detected, the comparison is unmarked (alpha = 0.05). Comparisons yielding significant differences are represented by a line connecting the arrows above the respective bars (**p < 0.01).

We next increased the inoculum of *nef*-defective HIV by 10-fold (30,000 TCIU, 5.6 ng of p24^gag^) to assess the robustness of the *nef*-deleted phenotype. By two weeks, two mice infected with the LAINef*dd* virus exhibited delayed replication and relatively low levels of virus in peripheral blood compared to LAI at the same dose (Figure 4A, LAINef*dd*-5 and LAINef*dd*-7 versus LAI-4, LAI-5, and LAI-6). These two LAINef*dd*-infected mice exhibited similar replication kinetics to LAINef*dd*-1 and LAINef*dd*-3 in Figure 3A. One mouse (LAINef*dd*-6) did not show detectable viral replication in the blood for 8 weeks, but ultimately reached about 1.5 million copies of viral RNA/ml (Figure 4A). A similar delay in viral replication has been reported for a humanized Rag2^−/−^γ_c_^−/−^ mouse infected with YU-2 [39]. It should be noted that despite active viral replication in these three mice receiving 30,000 LAINef*dd* TCIU, no CD4^+^ T cell decline was observed in peripheral blood (Figure 4B). LAI at 30,000 TCIU gave a dramatic depletion of CD4^+^ T cells in blood by seven weeks (Figures 4A and B).

We then examined the levels of systemic CD4^+^ T cell depletion in the mice receiving this higher dose of virus. In the mice injected with LAI there was a dramatic depletion of CD4^+^ T cells in the lymph node, spleen, bone marrow, lung, liver and double positive thymocytes (Figure 4C). Importantly, CD4^+^ T cells were preserved in all the tissues analyzed from the mice infected with the *nef*-defective virus including the human thymic organoid (Figure 4C). These results demonstrate that, even with 10-fold higher inoculums, *nef*-deleted LAI is still highly attenuated in its ability to deplete T cells and thymocytes. However, the delay in viral replication observed in Figure 4A appeared to be less dramatic at this higher dose for LAINef*dd*-5 and LAINef*dd*-7 than for LAINef*dd* infection at 3000 TCIU (Figure 3A). The extreme delay in LAINef*dd*-6 will be considered in the Discussion.

### Infection of BLT mice with a high dose of LAINef*dd*

**Figure 5:**
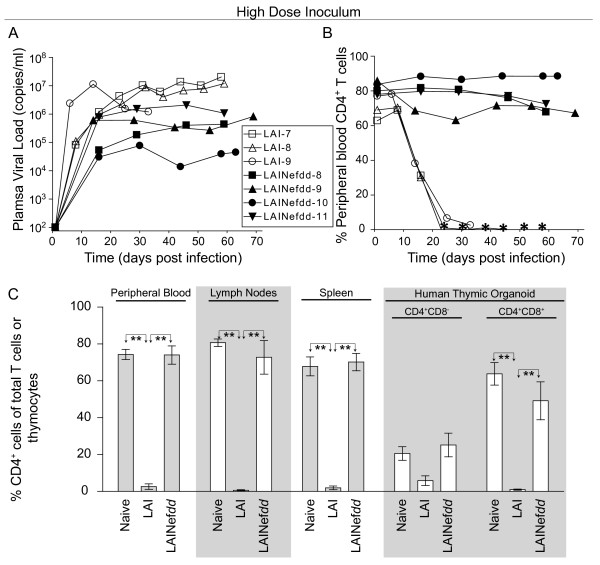
**Analysis of BLT humanized mice inoculated with a high dose of*nef*(−) or wild-type LAI. (A)** Each line depicts longitudinal plasma viral load data from individual BLT humanized mice infected with 600,000 TCIU of LAINef*dd* (closed symbols) or LAI (open symbols). All LAINef*dd* and LAI infected mice showed detectable viremia by two weeks post-exposure. **(B)** Each line depicts the percentage of CD4^+^ T cells in peripheral blood over time where each animal’s symbol is matched to the mice in (A). Mice infected with a high dose of LAINef*dd* showed minimal changes in CD4^+^ T cell percentages. In contrast, BLT humanized mice inoculated with an equal dose of LAI exhibited a very rapid decline of CD4^+^ T cells. Asterisks indicate that essentially all of these cells are eventually depleted in BLT humanized mice infected with 600,000 TCIU of LAI. **(C)** Naïve BLT mice (n = 5 in PB, spleen and HTO or n = 4 in LN) and BLT mice inoculated with 600,000 TCIU of LAINef*dd* (n = 4) exhibited similar levels of CD4^+^ cells, while mice inoculated with the same dose of LAI (n = 3) exhibited a drastic reduction in these cells. Shown are the percentages of human CD4^+^ T cells present in peripheral blood, lymph nodes and spleen, as well as the percentages of CD4^+^CD8^-^ and CD4^+^CD8^+^ thymocytes in the human thymic organoid. The percent of CD4^+^ T cells in peripheral blood or tissues was relative to total CD3^+^ T cells while the percent of CD4^+^CD8^-^ and CD4^+^CD8^+^ thymocytes was relative to total thymocytes. One-way ANOVA with three Bonferroni multiple comparisons tests was performed to compare the results within each tissue. If no difference was detected, the comparison is unmarked (alpha = 0.05). Comparisons yielding significant differences are represented by a line connecting the arrows above the respective bars (**p < 0.01).

To further assess the phenotype of *nef*-defective virus we tested the possibility that an additional 20-fold increase in viral inoculum would overwhelm the *nef*-deleted phenotype and result in pathogenic infection. A total of ten BLT mice (LAINef*dd*-8 through LAINef*dd*-17) were infected at this supraphysiological dose (6X10^5^ TCIU, 112 ng of p24^gag^). Two different outcomes were observed. With seven of these infections, there were at most small drops in the levels of CD4^+^ T cells in peripheral blood; but with three mice substantial drops in CD4^+^ T cells were observed (Figure 5B6B and 6D). As shown in Figure 5A and B, inoculation of BLT mice with 6 × 10^5^ TCIU of the wild-type LAI resulted in the rapid appearance of high levels of virus in peripheral blood, and depletion of peripheral blood CD4^+^. CD8^+^ T cells were also depleted as seen in late stages of HIV-1 infection (not shown, [40,41]). Viral replication in two of the LAINef*dd* mice yielded relatively high viral loads between 10^5^ and 10^6^ at two weeks (LAINef*dd*-9 and LAINef*dd*-11). In two other mice, plasma virus was less than 100,000 at two weeks (LAINef*dd*-8 and LAINef*dd*-10). At these levels of LAINef*dd* replication, CD4^+^ T cell loss was essentially absent (Figure 5B).

**Figure 6:**
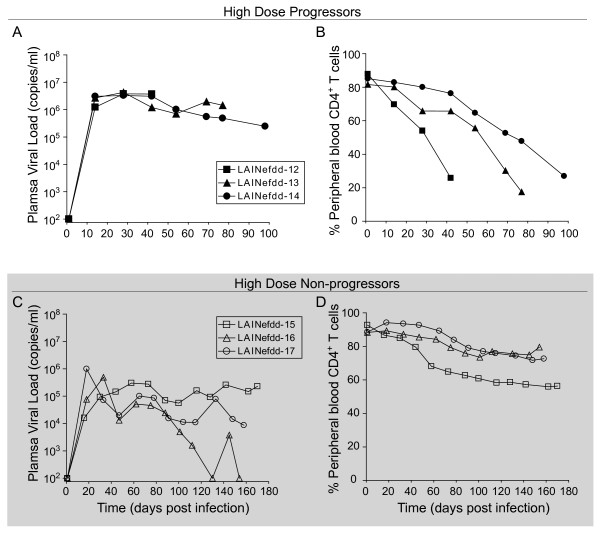
**Distinct phenotypes in BLT humanized mice during long term infection with a high dose of LAINef*dd***. **(A-B)** Each line depicts longitudinal plasma viral load data **(A),** or the CD4^+^ T cell percentages **(B)** from individual BLT humanized mice infected with 600,000 TCIU of LAINef*dd* where each animal in **(A)** and **(B)** is symbol matched. Persistent viral replication and loss of CD4^+^ T cells in the peripheral blood were observed in each of these mice. **(C-D)** Each line depicts longitudinal plasma viral load data **(C)** or the CD4^+^ T cell percentages **(D)** from individual BLT humanized mice infected with 600,000 TCIU of LAINef*dd* where each animal in **(C)** and **(D)** is symbol matched. Viral replication and minimal loss of CD4^+^ T cells in the blood were observed in these mice over this extended experimental analysis.

At eight weeks post infection, we sacrificed the four LAINef*dd*- and three LAI-infected mice to determine the levels of CD4^+^ T cells in tissues (Figure 5C). Systemically, infection with wild type LAI at this high dose resulted in near total depletion of CD4^+^ T cells in all tissues analyzed and double-positive thymocytes as well. Interestingly, single positive CD4^+^ thymocytes did not appear to be fully depleted by LAI (Figure 5C). Mice infected with LAINef*dd* at this higher dose, maintained normal levels of CD4^+^ T cells and double-positive thymocytes. These results demonstrate that over a 200-fold range in the dose of virus, Nef is necessary for rapid loss of CD4^+^ T cells and CD4^+^CD8^+^ thymocytes. A role for Vpr, Env or possibly other HIV-1 proteins is not excluded, but all of these genes are functional in LAI and LAINef*dd*[42-44]. A recent report found no impact of deleting *vpu* on CD4^+^ T cell depletion by HIV-1_AD8+_ in humanized mice [45].

### Infection of BLT mice with a high dose of LAINef*dd* for an extended time course

**Figure 7:**
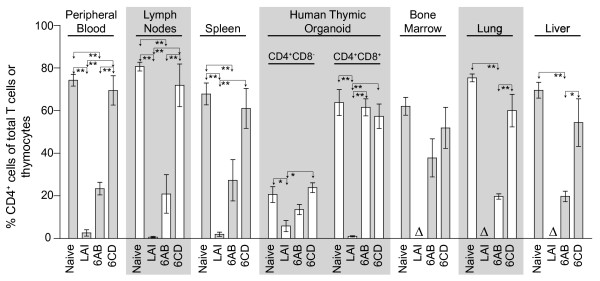
**Impact of long-term infection with a high dose of LAINef*dd*in BLT humanized mice.** The six BLT humanized mice infected with 600,000 TCIU of LAINef*dd* presented in Figure 6 were harvested for multiple tissue analyses at the last time points depicted in that figure. These mice are referred to here by the panels in which they appear in that figure (6AB or 6CD). Data from these mice are presented alongside data from naïve BLT humanized mice (n = 5 in PB, spleen and HTO or n = 4 in LN, BM, lung and liver) and BLT humanized mice inoculated with 600,000 TCIU of LAI (n = 3) to reveal that the CD4^+^ T cell loss patterns observed in the peripheral blood are mirrored by the multiple organ analyses performed. Shown are the percentages of human CD4^+^ T cells present in peripheral blood, lymph nodes, spleen, bone marrow, lung and liver, as well as the percentages of CD4^+^CD8^-^ and CD4^+^CD8^+^ thymocytes in the human thymic organoid. Delta symbols serve to indicate no data are available from bone marrow, lung or liver for LAI at 600,000 TCIU. The percent of CD4^+^ T cells in peripheral blood or tissues was relative to total CD3^+^ T cells while the percent of CD4^+^CD8^-^ and CD4^+^CD8^+^ thymocytes was relative to total thymocytes. One-way ANOVA with six Bonferroni multiple comparisons tests was performed to compare the results within each tissue. If no difference was detected, the comparison is unmarked (alpha = 0.05). Comparisons yielding significant differences are represented by a line connecting the arrows above the respective bars (*p < 0.05; **p < 0.01).

Our findings indicated that the dependence on Nef for replication was clearly evident at low and intermediate inoculums, but in some cases of the high initial inoculums of LAINef*dd* replication approached that of wild-type LAI. At high inoculum, the non-pathogenic phenotype of LAINef*dd* was still dramatic. We considered the possibility that longer courses of sustained infection may result in substantial CD4^+^ T cell depletion. For this purpose, we followed six LAINef*dd*-infected mice infected with 6.0X10^5^ TCIU for up to 170 days. As indicated above, three mice exposed to this high dose of LAINef*dd* exhibited reductions in CD4^+^ T cell levels in peripheral blood. These three mice had uncharacteristically high viral loads (>10^6^ copies/ml of plasma) by two weeks (Figure 6A). Associated with this high viral load was a reduction of CD4^+^ T cells in the peripheral blood (Figure 6B). However, despite viral loads near that of wild-type virus, the time courses of CD4^+^ T cell depletion were significantly delayed for an average of ten weeks for the *nef*-defective virus compared to less than three weeks for LAI (Figure 5B and 6B, Mantel-Cox Test, *p* = 0.025). The reduced levels of CD4^+^ T cells in peripheral blood were mirrored by similar reductions in the lymph node, spleen, lung and liver but not in bone marrow of these animals (Figure 7, see bars designated *6AB*). In contrast, the levels of thymocytes in these mice were not significantly reduced (Figure 7, *CD4*^*+*^*CD8*^*-*^*, CD4*^*+*^*CD8*^*+*^*, 6AB*). The observations from these three mice suggest that even in the case of clear reductions CD4^+^ T cells, the *nef*-deleted virus was still much less cytotoxic than the wild-type virus to thymocytes.

We considered the possibility that LAINef*dd*-12, LAINef*dd*-13 and LAINef*dd*-14 exhibited T cell depletion because rearrangements of the remnants of *nef* coding sequence may generate a severely truncated *nef* open reading frame with some restoration of function. In Additional file 3: Figure S3, we have aligned the sequences for the *nef* regions obtained by RT-PCR from the terminal bleed of these mice. All three of the sequences are identical to the sequence of the input virus. Therefore, *nef* from these three mice remained non-functional.

In Figure 6C and D, we present the final three mice that were infected with the highest dose of LAINef*dd* and were followed for 5–6 months post infection. The viral loads for these mice were maintained below 10^6^ copies LAINef*dd* RNA/ml blood throughout the experiment (Figure 6C). For all of these mice, we observed only small drops in peripheral blood CD4^+^ T cells (Figure 6D). The results are in contrast to the complete loss of CD4^+^ T cells observed as early as twenty-one days in mice infected with 600,000 TCIU of LAI and further establish the significantly attenuated pathogenic phenotype of LAINef*dd*. Consistent with the results in Figure 6D for peripheral blood, the long term infections also did not result in CD4^+^ T cell depletion in any of the organs analyzed- lymph node, spleen, bone marrow, lung, and liver (Figure 7, *6CD*). Single and double positive thymocytes were also clearly resistant to depletion by LAINef*dd* infection (Figure 7, *6CD*). In summary, the results indicate that the attenuated pathogenic phenotype of LAINef*dd* is not overwhelmed by a very high dose of virus.

### The relationship between viral load, T cell activation, and the loss of peripheral blood CD4^+^ T cells and thymocytes

Recent studies of HIV-1 infection in BLT mice and patients have found evidence of elevated CD38^+^HLA-DR^+^ CD8^+^ T cells in blood [46,47]. We also observed this effect with LAI infection. In naïve mice, 0.60% ± 0.11%, (n = 5) of CD 8^+^ T cells in blood were CD38^+^HLA-DR^+^. For LAI and LAINef*dd* infected mice, there were 8.5% ± 2.4% (n = 5) and 8.4% ± 2.6% (n = 3) CD38^+^HLA-DR^+^ CD8^+^ T cells in blood, respectively, which is significantly higher than for naïve mice (Mann Whitney test, *p* = 0.012 and *p* = 0.036). LAI and LAINef*dd* infected mice did not exhibit statistically different CD8^+^ T cell activation (*p* = 0.96). One explanation of these results is that relatively low levels of viral replication will induce T cell activation, but not T cell killing. In Table 1, we present the peak values for all twenty-six infected mice. All nine mice infected with LAI had peak viral loads of greater than 3X10^6^ copies of LAI RNA in blood with a mean ± S.E.M. of 8.2 × 10^6^ ± 1.8 × 10^6^ and a range of 3.6 to 20.4X10^6^ copies of viral RNA/ml. Only three of seventeen LAINef*dd* mice had peak viral loads >3X10^6^ copies of viral RNA (LAINef*dd*-12, 13, and 14, Figure 6A), and all three exhibited T cell loss (Figure 6B). LAINef*dd*-2,-4,-6,-11 had peak viral loads of 1-2 × 10^6^ ,but failed to deplete peripheral blood T cells (Table 1, Figure 3A4A and 5A). Therefore, peak viral loads >3,000,000 is associated with T cell depletion (Table 1*T cell Depletion*). CD4^+^CD8^+^ thymocyte depletion was not associated with peak viral load, but instead was determined by the presence or absence of Nef expression (Table 1*CD4*^*+*^*CD8*^*+*^*Depletion*).

**Table 1:** **Relationship between Peak Viral Load, T cell depletion and CD4**^**+**^**CD8**^**+**^**Thymocyte Depletion**^**a**^

**Mouse**	**Peak Viral Load**^**b**^**(highest to lowest)**	**Inoculum (TCIU)**^**c**^	**T cell Depletion**^**d**^	**CD4**^**+**^**CD8**^**+**^**Depletion**^**e**^
LAI-7	2.04 × 10^7^	600,000	Yes	Yes
LAI-8	1.19 × 10^7^	600,000	Yes	Yes
LAI-9	1.13 × 10^7^	600,000	Yes	Yes
LAI-4	6.70 × 10^6^	30,000	Yes	Yes
LAI-2	5.63 × 10^6^	3,000	Yes	Yes
LAI-6	4.99 × 10^6^	30,000	Yes	Yes
LAI-3	4.64 × 10^6^	3,000	Yes	Yes
LAI-1	4.25 × 10^6^	3,000	Yes	Yes
LAINef*dd*-13	4.24 × 10^6^	600,000	Yes	No
LAINef*dd*-12	3.77 × 10^6^	600,000	Yes	No
LAI-5	3.59 × 10^6^	30,000	Yes	Yes
LAINef*dd*-14	3.29 × 10^6^	600,000	Yes	No
LAINef*dd*-11	2.07 × 10^6^	600,000	No	No
LAINef*dd*-6	1.56 × 10^6^	30,000	No	No
LAINef*dd*-4	1.25 × 10^6^	3,000	No	No
LAINef*dd*-2	1.02 × 10^6^	3,000	No	No
LAINef*dd*-17	9.93 × 10^5^	600,000	No	No
LAINef*dd*-9	8.82 × 10^5^	600,000	No	No
LAINef*dd*-16	4.90 × 10^5^	600,000	No	No
LAINef*dd*-8	4.36 × 10^5^	600,000	No	No
LAINef*dd*-15	3.00 × 10^5^	600,000	No	No
LAINef*dd*-1	2.31 × 10^5^	3,000	No	No
LAINef*dd*-3	2.20 × 10^5^	3,000	No	No
LAINef*dd*-7	1.91 × 10^5^	30,000	No	No
LAINef*dd*-10	7.73 × 10^4^	600,000	No	No
LAINef*dd*-5	6.49 × 10^4^	30,000	No	No

^a^ Summary of viral loads at peak viremia and depletion of T cell and double positive thymocytes.

^b^ Copies viral RNA/ml plasma. LAI, 8.15 × 10^6^ ± 1.83 × 10^6^ (n = 9); LAINef*dd*, 1.24 × 10^6^ ± 0.32 × 10^6^ (n = 17).

^c^ Dose of intravenous inoculum presented in tissue culture infectious doses (TCIU).

^d^ “Yes,” mice exhibiting reduction of peripheral blood CD4^+^ T cells below 50% of pre-infection level; viral load, 7.06 × 10^6^ ± 1.47 × 10^6^ (n = 12). “No,” mice exhibiting peripheral blood CD4^+^ T cells not reduced below 50%; viral load, 0.70 × 106 ± 0.16 × 10^6^ (n = 14).

^e^ “Yes,” CD4^+^CD8^+^ thymocytes reduced below 10% total thymocytes. Viral loads for “Yes” and “No” same as LAI and LAINef*dd*, respectively (^b^).

A combined mechanism of T cell killing brought about by high viral replication plus reduced replacement of T cells by the thymus may account for the dramatic loss of T cells we have observed with LAI infection. In the case of the three LAINef*dd*-infected mice that lost peripheral blood T cells, there was no loss of CD4^+^CD8^+^ thymocytes which suggests that T cell replenishment may have been ongoing (Figure 7). This could account for the extended time course of T cell depletion for LAINef*dd*-12, LAINef*dd*-13 and LAINef*dd*-14 relative to that observed for LAI infections at the high viral dose (Figures 5B and 6B).

HIV-1 infection results in depression of thymic output of T cells [48]. We have determined that deletion of the *nef* coding sequence prevents thymocyte loss by even a high dose of *nef*-defective virus. For T cell depletion, the cytoxcity of LAI appears to depend on reaching a threshold of replication not usually achieved by LAINef*dd*. Our results are consistent with Nef indirectly depleting T cells by elevating HIV-1 replication. However, CD4^+^CD8^+^ thymocytes appear to be depleted by a different mechanism in which Nef is acting as a pathogenic factor.

## Discussion

Recent reports have demonstrated the utility of the BLT humanized mouse model for investigating HIV-1 infection [24,27,33,43,47]. HIV-1 establishes a systemic infection with high viral loads that exhibits pathogenic properties by depleting human CD4^+^ T cells. Of particular interest to us was the extremely cytopathic phenotype of CXCR4-tropic viruses like HIV-1_LAI_. This well characterized and fully functional virus replicates to high levels in BLT mice and rapidly depletes CD4^+^ T cells from blood and tissues [27]. The dramatic nature of this phenotype allowed us to investigate the role of Nef in two decisive phenotypes for the development of AIDS: maintenance of high levels of virus replication and depletion of CD4^+^ T cells and thymocytes [2,49]. Our studies show that at low inoculums the *nef*-deleted HIV-1_LAI_ was doubly defective with delayed replication and a severely blunted capacity to deplete CD4^+^ T cells and thymocytes. Mice infected with 3000 TCIU of wild-type LAI exhibited a viral load of 10^5^ copies/ml of RNA 14 days post inoculation, but LAINef*dd*-infected mice only reached a viral load of 10^5^ copies/ml at approximately 40, 40, 60, and 75 days post intravenous infection (Figure 3A). In addition, the low dose LAI-infected mice gave a 7-fold higher average peak viral load than the low dose LAINef*dd*-infected mice (Table 1). The pathogenic effect of Nef was also clearly demonstrated in Figure 3B, as LAI infection (3000 TCIU) resulted in an almost complete depletion of CD4^+^ T cells from peripheral blood, but LAINef*dd* infection resulted in at best a modest reduction in CD4^+^ T cells levels even after viral loads became elevated. Similar results were obtained at the ten-fold higher dose of 30,000 TCIU of LAI and LAINef*dd* (Figure 4). Thus, Nef is important, not only for efficient viral propagation, but also full pathogenicity in vivo.

At 600,000 TCIU of LAINef*dd*, CD4^+^ T cells were not depleted in seven of ten mice despite peak viral loads of 0.75 × 10^6^ ± 0.25 × 10^6^ demonstrating the great importance of Nef for pathogenicity. However, three of ten mice infected with the highest dose of *nef*-deleted virus were observed to have a pathogenic phenotype (Figure 5B). These three mice exhibited efficient viral replication as reflected by high peak viral loads (3.8 × 10^6^ ± 0.2 × 10^6^), but delayed reductions in CD4^+^ T cell levels. We attribute the loss of T cells in these mice to the high levels of replication of LAINef*dd* since no repair of the *nef* reading frame was observed. Repair of the defective *nef* was not expected, as all *nef*-defective HIV-1s from patients develop expanded deletions to include most of the *nef* coding sequence while sparing the polypurine tract [4,7-9,36].

Precedent for a delayed pathogenic phenotype for *nef*-deleted HIV-1 has been reported for patients infected with *nef*-deleted virus that were followed for over 15 years (Table 2). Three of eight patients lost CD4^+^ T cells to the extent that anti-retroviral therapy became necessary. The remaining five patients appear to be elite controllers (Table 2). That 63% of patients infected with *nef*-deleted HIV-1 that were documented for a sufficient length of time are elite controllers is in stark contrast to the less than 1% of elite controllers found in patients infected with *nef*-intact HIV-1 [1]. A simple interpretation is that the patient’s immune system effectively controlled the *nef*-deleted HIV-1, but in three patients where the *nef*-defective virus was able to maintain replication, CD4^+^ T cell levels began to decline after many years [35,50]. Unfortunately, thymic function in these three patients is not available.

**Table 2:** **Outcomes for patients infected with HIV-1*nef*(−) for longer than 15 years**^**a**^

**Patient Identifier**	**Viral Load**^**b**^	**Date Infected**	**ART**^c^
D36	Low	1980	Yes, 1999
C54	Low	1984	No
C98	Low	1982	Yes, 1999
Patient 1	BLD	Prior to 1983	Yes, 1998
Patient SG1	BLD	1985	No
C49	BLD	1984	No
C64	BLD	1983	No
C135	BLD	1981	No

^a^ Patients reported to be infected by *nef*(−) HIV-1 for over fifteen years are presented with the appropriate identifier. D36, C54, C98, C49, C64, and C135 are from the Sydney Blood Bank Cohort [5,50,51]. Patient 1 was reported by Sullivan and co-workers [6,36,52]. Patient SG1 was reported by Benedetto and co-workers [9,35]. ^b^*Viral Load* represented as “Low” indicates copies of viral RNA were generally found to be less than 10,000 copies/ml of peripheral blood but detectable. “BLD,” indicates viral load generally below the level of detection. D36, C54 and C98 had viral loads that were consistently detectable [50,51]. Patient 1, Patient SG1, C49, C64, and C135 viral loads were not detectable [6,35,50]. The last four patients may have suppressed their HIV-1 infections by vigorous cytotoxic T cell responses [35,51]. C54 did not appear to be progressing to AIDS at the time of death in 2001 [50]. ^c^ART is antiretroviral therapy.

Several Nef activities may be critical factors in the attenuation of LAI pathogenicity and replication that we observed. For example, we observed considerable delay in appearance of viral RNA in the blood for the lowest dose (Figure 3A). The loss of the enhancement of virion infectivity function could be significant early in infection to slow the rate of spread of the virus [37,38]. Subsequently, following the delay in replication, higher levels of virus could reduce the impact of this effect and result in some LAINef*dd*-infected mice presenting with viral loads over 10^6^. It is also important to determine the importance of another Nef activity, CD4 downregulation. Previous results from infections of SCID-hu thy/liv implants with Nefs mutated for residues (W57L58) critical for CD4 downregulation have given conflicting results. As a result, the importance of CD4 downregulation for replication and pathogenicity *in vivo* is unresolved [21,22,53]. The results presented here demonstrate that BLT mice have the potential to serve as an excellent model to address the roles of specific Nef functions described *in vitro* on Nef’s phenotype *in vivo*.

We did not observe loss of CD4^+^CD8^+^ thymocytes in LAINef*dd* infections despite virtually complete depletion of this same cell population by LAI. Further, it appeared that CD4^+^CD8^-^ thymocytes were resistant to killing by LAI. This *in vivo* result is similar to the ex vivo result reported by Choudhary *et al*. who reported a greater cytotoxic effect by CXCR4-trophic HIV-1 on CD4^+^CD8^+^ than on CD4^+^CD8^-^ thymocytes [31]. This effect was attributed to CD4^+^CD8^+^, but not CD4^+^CD8^-^ thymocytes, being primed for negative selection. Potentially consistent with the proposed susceptibility of CD4^+^CD8^+^ thymocytes to apoptosis, Nef has been reported to induce PD-1 and FasL [54,55].

In summary, we have demonstrated that LAI lacking Nef function is greatly attenuated for pathogenicity. However, under supraphysiological conditions of infection, this linkage is not absolute; and the defective virus retains some capacity to systemically reduce CD4^+^ T cells. HIV-1 gene products other than Nef that may be responsible for the residual pathogenicity of LAINef*dd* are Vpr or Env [42,43,56]. The availability of an *in vivo* system to study the roles of HIV-1 accessory genes in viral replication and depletion of CD4^+^ T cells and thymocytes will help address currently unresolved questions. In particular, the mechanism of Nef induced killing of CD4^+^CD8^+^ thymocytes needs to be explored in future studies as there is evidence of an important role for HIV-1 inhibition of T cell replenishment from the thymus in disease progression [32,57].

## Conclusion

We have investigated the infection of BLT humanized mice by wild-type and *nef*-defective HIV-1. With wild type virus, there is rapid and near total depletion of CD4^+^ T cells in blood and in multiple tissue compartments. CD4^+^CD8^+^ thymocytes are also rapidly destroyed. With *nef*-defective virus, the course of infection is dramatically different. Despite the establishment of robust viral replication, there is rarely much loss of CD4^+^ T cells over the time frame that the wild-type virus systemically depletes these cells. With a high initial inoculum and an extended time course of infection, *nef*-defective virus may exhibit pathogenic properties. These observations are consistent with the conclusion that Nef is a replication factor and indirectly accelerates T cell killing by inducing high levels of viral replication. This mechanism does not apply to CD4^+^CD8^+^ thymocytes, and our results suggest that Nef has a direct role in thymocyte killing. The effectiveness of CXCR4-trophic HIV-1 in depleting CD4^+^ T cells may be enhanced by killing thymocytes and preventing thymic T cell replenishment.

## Methods

### Preparation of humanized BLT mice

Humanized BLT mice were prepared as previously described [24-27,34,58-60]. Briefly, thymus/liver implanted NOD/SCID or NOD/SCID IL-2γ^−/−^ mice (The Jackson Laboratories, Bar Harbor, ME) were transplanted with autologous human CD34^+^ cells isolated from fetal liver (Advanced Bioscience Resources, Alameda, CA). Human reconstitution in the peripheral blood of these mice was monitored periodically by flow cytometry (FACSCanto; BD Biosciences). Mice were maintained either at the Animal Resources Center, UT Southwestern Medical Center at Dallas (UTSWMC) or at the Division of Laboratory Animal Medicine, University of North Carolina at Chapel Hill (UNC-CH) in accordance with protocols approved by the UTSWMC or UNC-CH Institutional Animal Care and Use Committee. To ensure genetic diversity, seventeen different tissue donors were used to generate three groups of mice used for the experiments presented in this manuscript. Specifically, the percent engraftment for five naïve mice was 49.8% ± 13.9% human CD45^+^ cells in blood from five of the donors, for the nine LAI-infected mice it was 40.3% ± 8.0% human CD45^+^ cells in blood from seven of the donors, and for seventeen LAINef*dd*-infected mice it was 51.4% ± 4.8% human CD45^+^ cells in blood from nine of the donors. Engraftment did not significantly differ between the groups. Peak level of virus in blood did not correlate with human cell engraftment.

### Cell lines and culture conditions

293T and TZM-bl cells were maintained in Dulbecco’s modified Eagle’s medium (DMEM; Cellgro, Herndon, VA) supplemented with 10% fetal bovine serum (FBS; Cellgro), 100 IU/ml of penicillin, 100 μg/ml streptomycin, and 2 mM glutamine (Cellgro) in 10% CO_2_ at 37°C.

### Proviral clones

The molecular clone, LAI (accession # K02013), is described by Peden *et al.*[29]. pLAINef*dd* was made by first removing the internal XhoI and Acc65I fragment internal to *nef*, treating with Klenow and religating. This removes nucleotides 8495–8608, inclusive. The deleted LAI provirus was then cut at the reconstructed XhoI site, treated with Klenow and religated to introduce a frame shift (four base insertion) in addition to the deletion 114 bases. Second, nucleotides, 8692–8981, inclusive, were deleted by a site-directed mutagenesis strategy (Stratagene, USA). LAINefXhoI was constructed to be defective for *nef* by cutting the provirus with the single cutter XhoI, filling in with Klenow and religating. This leaves *nef* coding sequence intact but introduces a four-base frameshift at *nef* codon 35. Lack of Nef expression by the mutants was confirmed by Western blot analysis. Our LAI construct is comparable to the double deleted NL4-3 construct of Gibbs *et al*. [61]. Converting NL4-3 numbers to homologous LAI numbers to compare the two constructs: the deletions were NL4-3/LAI (8428/8495 to 8650/8608 for the 5′ deletion and 8718/8692 to 8983/8981 for the 3′ deletion). This NL4-3 construct was used to produce virus for SCID-hu studies [22].

### Virus production, exposure of BLT mice to HIV-1_LAI_ or *nef*-deleted HIV-1_LAI,_ tissue harvesting and cytometric analyses

Stocks of LAI, LAINef*dd* and LAIXhoI were prepared and titered as we previously described [62,63]. Briefly, proviral clones were transfected into 293T cells. Viral supernatant was collected 48 hours after transfection and diluted in Dulbecco’s modified Eagle’s medium (DMEM) supplemented with 10% fetal bovine serum, 100 IU penicillin/ml, 100 μg/ml streptomycin, and 2 mM glutamine. TZM-bl cells were infected in 12-well tissue culture plates with 0.4 ml of virus containing medium for two hours. Then 1.0 ml of supplemented DMEM was added, and the plates were incubated overnight. Virus containing medium was removed the next day and the incubation was continued for 24 hours. The cells were fixed and stained (40 hours after first exposure to virus). The titers of these viral stocks were determined in triplicate and at least two different titer determinations were performed in each batch of virus used for all the experiments described in this manuscript. p24^gag^ was determined for each prep of virus by ELISA.

Intravenous exposure of BLT mice with HIV-1_LAI_ or HIV-1_LAI_*dd* was conducted via tail vein injection with 3000, 30,000 and 6 × 10^5^ tissue culture infectious units (TCIU). Viral load in peripheral blood of infected mice was monitored longitudinally by quantitative real-time PCR using Taqman RNA to-C_T_^™^ 1-step kit from Applied Biosystems, USA [34,64]. The sequences of the forward and reverse primers and the Taqman probe for PCR were: 5′- CATGTTTTCAGCATTATCAGAAGGA- 3′, 5′-TGCTTGATGTCCCCCCACT- 3′, and 5′- FAM CCACCCCACAAGATTTAAACACCATGCTAA- Q- 3′ , respectively. CD4^+^ and CD8^+^ T cell levels were monitored by flow cytometric analysis.

Flow cytometric immunophenotyping was performed on peripheral blood samples longitudinally and mononuclear cells were isolated from tissues at harvest. Whole peripheral blood from humanized mice was analyzed according to the BD Biosciences Lyse/Wash protocol (Cat. No. 349202) as we have previously described [24,26,27]. Briefly, following antibody labeling of whole blood, red blood cells were lysed. The remaining cells were washed, fixed; and the sample was analyzed by flow cytometry. Tissue mononuclear cell isolations and immunophenotyping analyses were also performed according to published methods [24,26,27]. Flow cytometric gating for all samples was performed as follows: (step 1) forward and side scatter properities were utilized to set a live cell gate; (step2) live cells were then analyzed for expression of the human pan-leukocyte marker CD45; (step 3) human leukocytes were then analyzed for hCD3, hCD4 and/or hCD8 expression (step 4) in the case of CD8^+^ T cell activation analyses, peripheral blood human CD8+ T cells were analyzed for hCD38 and HLA-DR expression.

### Sequence analysis of plasma virions

Viral RNA was extracted from 20 μl of plasma from infected mice using the QIAamp Viral RNA Mini kit (Qiagen Sciences, USA). RNA was then reverse transcribed into cDNA, which was then subject to nested PCR. The outer primers for *nef* amplification are: 5′-AGCTTGCTCAATGCCACAGCC-3′ and 5′-GCTGCATATAAGCAGCTGCTTTTTG-3′. And the inner primers are: 5′-TAGAGCTATTCGCCACATACC-3′ and 5′-GCTTGCTACAAGGGACTTTCCGC-3′. Gel purified PCR products were sequenced and the sequences aligned to HIV_LAI_ sequences to determine if sequence changes had occurred.

### Statistics

Log-rank (Mantel-Cox) tests (alpha level, 0.05) for initial viremia detection were performed using survival analyses in Prism version 4 (GraphPad). Unpaired two-tailed *t* tests and one-way ANOVA were also performed in Prism version 4 (Graph Pad). All data were plotted as mean ± S.E.M.

## Competing interests

The authors declare that they have no competing interests.

## Authors’ contributions

WZ, PWD, RLW, JFK, TN, and ML performed experiments and made Figures. PWD, JVG and JLF designed experiments and wrote the manuscript. All authors read and approved the final manuscript.

## Supplementary Material

Additional file 1 **Figure S1. Alignment of LTR sequence from HIV-1**_**LAI**_**and Sydney Blood Bank Cohort Patient D36.***LAI LTR* is the nucleotide sequence of LAI U3, R and U5. U3 (black, red and green) and is numbered from the transcription start site to the 5′ end of U3 (−1 to −454). R (brown) is numbered from +1 to +97 and U5 (orange) is numbered from +98 to +181. Above LAI LTR sequence is the translation of *nef* in three-letter amino acid code (black). The three colors for LAI U3 nucleotide sequence indicate- U3 general sequence (black), U3 sequence that is deleted in LAINef*dd* (red) and U3 sequence that represents transcription factor binding sites (green). *D36 LTR* is a nucleotide sequence from the donor in the Sydney Blood Bank Cohort (NCBI Accession DQ287276). The sequence is derived from a blood sample taken approximately 20 years post infection and is representative of *nef*s with large deletions on either side of the PPT [4,5]. D36 U3 sequence is presented in black and green. Black is general U3 sequence and green represents transcription factor binding sites. Dashes (black) represent D36 deleted sequence. Blue “X’s” are for reported upstream transcription factor binding sites that are deleted in D36 LTR sequence. Asterisks indicate identical residues between LAI and D36 LTRs. A four base insertion in D36 LTR between A(−95) and C(−94) is shown below the asterisks. D36 R and D36 U5 are brown and orange, respectively. The sequence for D36 U5 is not complete. The conserved U3 core promoter (−1 to −122) contains binding sites for multiple transcription factors. Estable et al. [65] determined that the conservation of the individual binding sites to be- TFIID (97%), three intact SP1 (95%) and two intact NF-κB (85%). Just upstream of the core promoter the Ras response element binding factor 2, RBF-2 (63%) and E26 transformation-specific domain protein, Ets (87%) also are conserved. Note that all of these sites (green) are intact in LAINef*dd* and are present in the D36 LTR with the exception of Ets and possibly RBF-2. Binding sites for upstream stimulatory factor (USF), nuclear factor of activated T cells (NFAT) and chicken ovalbumin upstream promoter transcription factor (COUP-TF) are deleted in LAINef*dd* and D36 (blue). Deletion of the USF palindromic binding site, CACGTG, was reported to enhance viral replication [66]. However, CACGTG is only 15% conserved and USF1 and USF2 overexpression activates HIV LTR independently of this site [65,67]. NFAT-1 binding was reported to negatively impact HIV-1 replication though later studies found no effect and demonstrated that NFAT-1 binds to the tandem NF-κB sites [66,68,69]. COUP-TF binds at the gapped palindrome GGTCAN_9_TGACC (LAI LTR nucleotides −348 to −320). This palindrome is found in LAI but is not conserved in subtype B *nef*s [70,71]. Click here for file

Additional file 2 **Figure S2. Infectivity of LAINefXhoI and LAINef*dd*are similar.** A single round infection assay was performed with the indicator cell line, TZM-bl, with LAI, LAINefXhoI or LAINef*dd*. The mean infectivity of LAI (10,200 ± 1270 TCIU per ng p24^gag^) was set at 100%. The infectivities of LAINefXhoI (5260 ± 630 TCIU per ng p24^gag^) and LAINef*dd* (3400 ± 570 per ng of p24^gag^) were significantly less than LAI but not different from each other. Comparisons yielding significant differences are represented by a line connecting the arrows above the respective bars (**p < 0.01). Click here for file

Additional file 3 **Figure S3. Alignment of*nef*sequences with LAI and LAINef*dd*sequences.***nef* was amplified by RT-PCR from blood of LAINef*dd*-12, LAINef*dd*-13 and LAINef*dd*-14 collected at tissue harvest (40, 78 and 90 days, respectively; Figure 6A and 6B). The amplified products were sequenced and aligned by Clustal X. Asterisks on the bottom line represent identical residues and dashes represent nucleotides deleted during the construction of the proviral clone of LAINef*dd*. Click here for file
