# Supplementary material for: Nef functions in BLT mice to enhance HIV-1 replication and deplete CD4+CD8+ thymocytes
Source: Retrovirology. 2012 May 28;9:44. doi: 10.1186/1742-4690-9-44 (PMC3403983; doi:10.1186/1742-4690-9-44)
Supplement: Additional file 1 — Figure S1. Alignment of LTR sequence from HIV-1LAIand Sydney Blood Bank Cohort Patient D36.LAI LTR is the nucleotide sequence of LAI U3, R and U5. U3 (black, red and green) and is numbered from the transcription start site to the 5′ end of U3 (−1 to −454). R (brown) is numbered from +1 to +97 and U5 (orange) is numbered from +98 to +181. Above LAI LTR sequence is the translation of nef in three-letter amino acid code (black). The three colors for LAI U3 nucleotide sequence indicate- U3 general sequence (black), U3 sequence that is deleted in LAINefdd (red) and U3 sequence that represents transcription factor binding sites (green). D36 LTR is a nucleotide sequence from the donor in the Sydney Blood Bank Cohort (NCBI Accession DQ287276). The sequence is derived from a blood sample taken approximately 20 years post infection and is representative of nefs with large deletions on either side of the PPT [4,5]. D36 U3 sequence is presented in black and green. Black is general U3 sequence and green represents transcription factor binding sites. Dashes (black) represent D36 deleted sequence. Blue “X’s” are for reported upstream transcription factor binding sites that are deleted in D36 LTR sequence. Asterisks indicate identical residues between LAI and D36 LTRs. A four base insertion in D36 LTR between A(−95) and C(−94) is shown below the asterisks. D36 R and D36 U5 are brown and orange, respectively. The sequence for D36 U5 is not complete. The conserved U3 core promoter (−1 to −122) contains binding sites for multiple transcription factors. Estable et al. [65] determined that the conservation of the individual binding sites to be- TFIID (97%), three intact SP1 (95%) and two intact NF-κB (85%). Just upstream of the core promoter the Ras response element binding factor 2, RBF-2 (63%) and E26 transformation-specific domain protein, Ets (87%) also are conserved. Note that all of these sites (green) are intact in LAINefdd and are present in the D36 LTR with the exception of Ets [file 1742-4690-9-44-S1.pdf]

Figure S1.

```
           GluGlyLeuIleHisSerGlnArgArgGlnAspIleLeuAspLeuTrp
LAI LTR  TGGAAGGGCTAATTCACTCCCAACGAAGACAAGATATCCTTGATCTGTGG -405
D36 LTR  TGGAAGGGCTAATTCACTCCCAAAGAAGACAAGATA-------------- -189
         *********************** ************


         IleTyrHisThrGlnGlyTyrPheProAspTrpGlnAsnTyrThrProGl
LAI LTR  ATCTACCACACACAAGGCTACTTCCCTGATTGGCAGAACTACACACCAGG -355
D36 LTR  ------CACA------------------------------------XXXX -184
               ****

         yProGlyValArgTyrProLeuThrPheGlyTrpCysTyrLysLeuValP
LAI LTR  GCCAGGGGTCAGATATCCACTGACCTTTGGATGGTGCTACAAGCTAGTAC -305
D36 LTR  XXXXXXXXX COUP-TF XXXXXXXXXXXXXXXGTGCTGCAAACTATTAC -168
                                          ***** *** *** ***

         roValGluProAspLysValGluGluAlaAsnLysGlyGluAsnThrSer
LAI LTR  CAGTTGAGCCAGATAAGGTAGAAGAGGCCAATAAAGGAGAGAACACCAGC -250
D36 LTR  CAGTGGAGTCAGCGAAAATAGAAGAGGCCAACGGAGGAGAAAACAACAGA -118
         **** *** ***  **  *************   ****** **** ***

         LeuLeuHisProValSerLeuHisGlyMetAspAspProGluArgGluVa
LAI LTR  TTGTTACACCCTGTGAGCCTGCATGGAATGGATGACCCTGAGAGAGAAGT -205
D36 LTR  TTGXXXXXXXXXXXXXXXX NFAT-1 XXXXXXXXXXXXXXX-------- -115
         ***

         lLeuGluTrpArgPheAspSerArgLeuAlaPheHisHisValAlaArgG
LAI LTR  GTTAGAGTGGAGGTTTGACAGCCGCCTAGCATTTCATCACGTGGCCCGAG -155
D36 LTR  ----------------------------------XXX USF XXX-----

               /Ets\             /RBF-2 \
         luLeuHisProGluTyrPheLysAsnCysEnd                /_
LAI LTR  AGCTGCATCCGGAGTACTTCAAGAACTGCTGACATCGAGCTTGCTACAAG -105
D36 LTR  --------------------------TGCTGAT--------------TGG -105
                                   ******                 *

         NF-κB   \   / NF-κB   \    /   Sp1   \ /  SP1  \ /
LAI LTR  GGACTTTCCGCTGGGGACTTTCCAGGGAGGCGTGGCCTGGGCGGGACTGG -55
D36 LTR  GGACTTTCCACTGGGGACTTTCCAAGAAGGCGCGGCCTGGGCGGGACGGG -55
         *********^************** * ***** ************** **
                 TCCG

           SP1    \               /TFIID\
LAI LTR  GGAGTGGCGAGCCCTCAGATGCTGCATATAAGCAGCTGCTTTTTGCCTGT -5
D36 LTR  GGAGTGGCGAGCCCTCAGATGCTGCATATAAGCAGCTGCTTTCTGCCTGT -5
         ****************************************** *******


LAI LTR  ACTGGGTCTCTCTGGTTAGACCAGATTTGAGCCTGGGAGCTCTCTGGCTA +46
D36 LTR  ACTGGGTCTCTCTGGTTGGGCCAGATCTGAGCCTGGGAGCTCTCTGGCTA +46
         ***************** * ****** ***********************


LAI LTR  ACTAGGGAACCCACTGCTTAAGCCTCAATAAAGCTTGCCTTGAGTGCTTC +96
D36 LTR  ACTAGGGAACCCACTGCTTAAGCCTCAATAAAGCTTGCCTTGAGTGCTTC +96
         **************************************************


LAI LTR  AAGTAGTGTGTGCCCGTCTGTTGTGTGACTCTGGTAACTAGAGATCCCTC +146
D36 LTR  AAGTAGTGTGTGCCCGTCTGTTGTGTGACTCTGGTAACTAGAGATCCCTC +146
         **************************************************

LAI LTR  AGACCCTTTTAGTCAGTGTGGAAAATCTCTAGCAG                +181
D36 LTR  AGACC-TTTTAGTCAG                                   +161
         ***** **********
```

Figure S1. Alignment of U3 from LAI and Sydney Blood Banks Cohort Patient D36.
