# Supplementary figures and images for: Nef functions in BLT mice to enhance HIV-1 replication and deplete CD4+CD8+ thymocytes
Source: Retrovirology. 2012 May 28;9:44. doi: 10.1186/1742-4690-9-44 (PMC3403983; doi:10.1186/1742-4690-9-44)

Figure S2

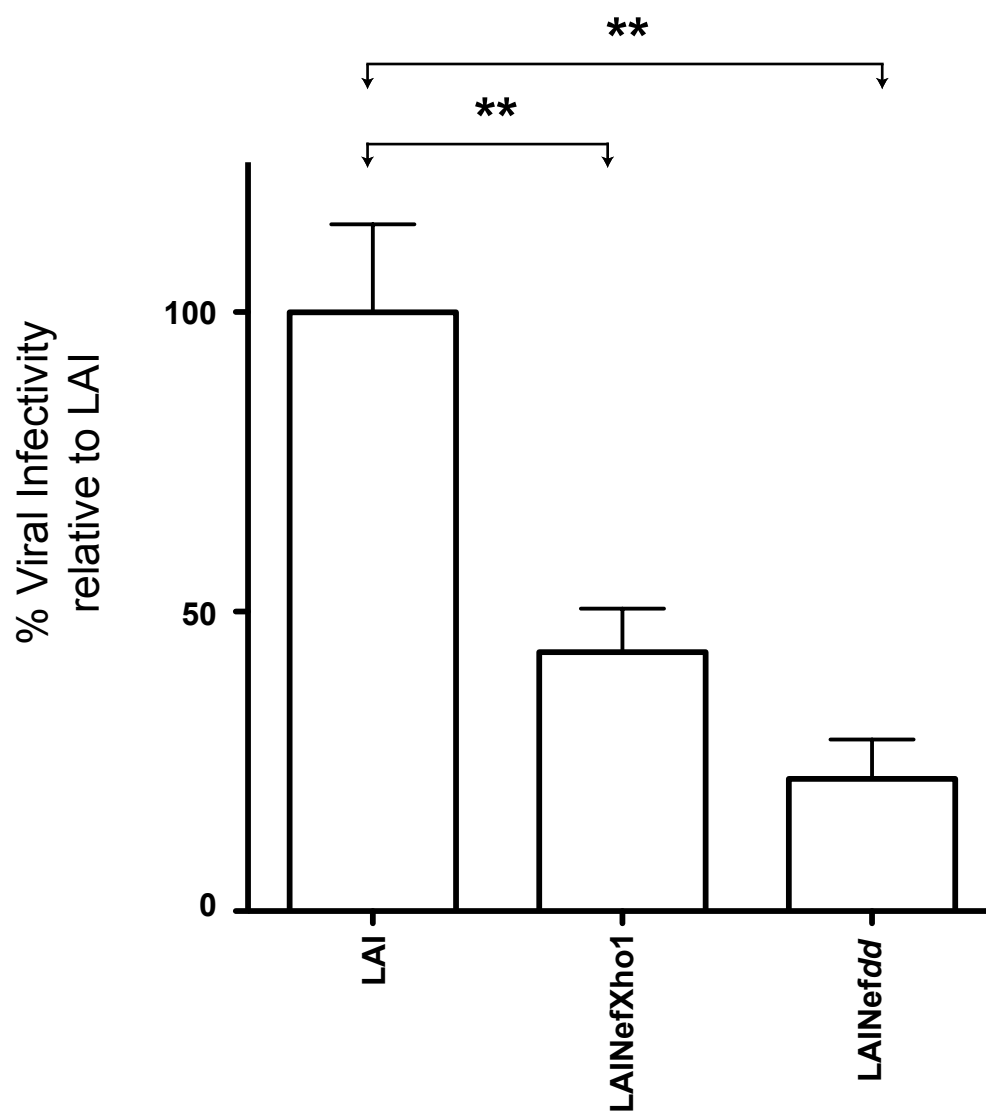

Figure S2. Infectivity between LAINefXho1 and LAINefdd are similar.

Supplement: Additional file 2 — Figure S2. Infectivity of LAINefXhoI and LAINefddare similar. A single round infection assay was performed with the indicator cell line, TZM-bl, with LAI, LAINefXhoI or LAINefdd. The mean infectivity of LAI (10,200 ± 1270 TCIU per ng p24gag) was set at 100%. The infectivities of LAINefXhoI (5260 ± 630 TCIU per ng p24gag) and LAINefdd (3400 ± 570 per ng of p24gag) were significantly less than LAI but not different from each other. Comparisons yielding significant differences are represented by a line connecting the arrows above the respective bars (**p < 0.01). [file 1742-4690-9-44-S2.pdf]
