# Supplementary material for: Nef functions in BLT mice to enhance HIV-1 replication and deplete CD4+CD8+ thymocytes
Source: Retrovirology. 2012 May 28;9:44. doi: 10.1186/1742-4690-9-44 (PMC3403983; doi:10.1186/1742-4690-9-44)
Supplement: Additional file 3 — Figure S3. Alignment ofnefsequences with LAI and LAINefddsequences.nef was amplified by RT-PCR from blood of LAINefdd-12, LAINefdd-13 and LAINefdd-14 collected at tissue harvest (40, 78 and 90 days, respectively; Figure 6A and 6B). The amplified products were sequenced and aligned by Clustal X. Asterisks on the bottom line represent identical residues and dashes represent nucleotides deleted during the construction of the proviral clone of LAINefdd. [file 1742-4690-9-44-S3.doc]

**Figure S3** (page 1 of 2)

1 MetGlyGlyLysTrpSerLysSerSerValValGlyTrpProThrValArgGluArgMet

LAINef*dd*-12 1 ATGGGTGGCAAGTGGTCAAAAAGTAGTGTGGTTGGATGGCCTACTGTAAGGGAAAGAATG

LAINef*dd*-13 1 ATGGGTGGCAAGTGGTCAAAAAGTAGTGTGGTTGGATGGCCTACTGTAAGGGAAAGAATG

LAINef*dd*-14 1 ATGGGTGGCAAGTGGTCAAAAAGTAGTGTGGTTGGATGGCCTACTGTAAGGGAAAGAATG

LAI 1 ATGGGTGGCAAGTGGTCAAAAAGTAGTGTGGTTGGATGGCCTACTGTAAGGGAAAGAATG

************************************************************

21 ArgArgAlaGluProAlaAlaAspGlyValGlyAlaAlaSerArgAspLeuLysAsnHis

LAINef*dd*-12 61 AGACGAGCTGAGCCAGCAGCAGATGGGGTGGGAGCAGCATCTCGA*tcga*-----------

LAINef*dd*-13 61 AGACGAGCTGAGCCAGCAGCAGATGGGGTGGGAGCAGCATCTCGA*tcga*-----------

LAINef*dd*-14 61 AGACGAGCTGAGCCAGCAGCAGATGGGGTGGGAGCAGCATCTCGA*tcga*-----------

LAI 61 AGACGAGCTGAGCCAGCAGCAGATGGGGTGGGAGCAGCATCTCGAGACCTGGAAAAACAT

*********************************************

41 GlyAlaIleThrSerSerAsnThrAlaAlaThrAsnLeuLeuCysAlaTrpLeuGluAla

LAINef*dd*-12 ------------------------------------------------------------

LAINef*dd*-13 ------------------------------------------------------------

LAINef*dd*-14 ------------------------------------------------------------

LAI 121 GGAGCAATCACAAGTAGCAATACAGCAGCTACCAATGCTGCTTGTGCCTGGCTAGAAGCA

61 GlnGluGluGluGluValGlyPheProValThrProGlnValProLeuArgProMetThr

LAINef*dd*-12 ---------------------------------------GTACCTTTAAGACCAATGACT

LAINef*dd*-13 ---------------------------------------GTACCTTTAAGACCAATGACT

LAINef*dd*-14 ---------------------------------------GTACCTTTAAGACCAATGACT

LAI 181 CAAGAGGAGGAGGAGGTGGGTTTTCCAGTCACACCTCAGGTACCTTTAAGACCAATGACT

*********************

81 TyrLysAlaAlaValAspLeuSerHisPheLeuLysGluLysGlyGlyLeuGluGlyLeu

LAINef*dd*-12 241 TACAAGGCAGCTGTAGATCTTAGCCACTTTTTAAAAGAAAAGGGGGGACTGGAAGGGCTA

LAINef*dd*-13 241 TACAAGGCAGCTGTAGATCTTAGCCACTTTTTAAAAGAAAAGGGGGGACTGGAAGGGCTA

LAINef*dd*-14 241 TACAAGGCAGCTGTAGATCTTAGCCACTTTTTAAAAGAAAAGGGGGGACTGGAAGGGCTA

LAI 241 TACAAGGCAGCTGTAGATCTTAGCCACTTTTTAAAAGAAAAGGGGGGACTGGAAGGGCTA

************************************************************

101 IleHisSerGlnArgAspGlnAspIleLeuAspLeuTrpIleTyrHisTheGlnGlyTyr

LAINef*dd*-12 A-----------------------------------------------------------

LAINef*dd*-13 A-----------------------------------------------------------

LAINef*dd*-14 A-----------------------------------------------------------

LAI 301 ATTCACTCCCAACGAAGACAAGATATCCTTGATCTGTGGATCTACCACACACAAGGCTAC

*

(page 2 of 2)

121 PheProAspTrpGlnAsnTyrThrProGlyProGlyValArgTyrProLeuThrPheGly

LAINef*dd*-12 ------------------------------------------------------------

LAINef*dd*-13 ------------------------------------------------------------

LAINef*dd*-14 ------------------------------------------------------------

LAI 361 TTCCCTGATTGGCAGAACTACACACCAGGGCCAGGGGTCAGATATCCACTGACCTTTGGA

141 TrpCysTyrLysLeuValProValGluProAspLyaValGluGlrAlaAsnLysGlyGlu

LAINef*dd*-12 ------------------------------------------------------------

LAINef*dd*-13 ------------------------------------------------------------

LAINef*dd*-14 ------------------------------------------------------------

LAI 421 TGGTGCTACAAGCTAGTACCAGTTGAGCCAGATAAGGTAGAAGAGGCCAATAAAGGAGAG

161 AsnThrSerLeuLeuHisProValSerLeuHisGlyMetAspAspProGluArgGluVal

LAINef*dd*-12 ------------------------------------------------------------

LAINef*dd*-13 ------------------------------------------------------------

LAINef*dd*-14 ------------------------------------------------------------

LAI 481 AACACCAGCTTGTTACACCCTGTGAGCCTGCATGGAATGGATGACCCTGAGAGAGAAGTG

181 LeuGluTrpArgPheAspSerArgLeuAlaPheHisHisValAlaArgGluLeuHisPro

LAINef*dd*-12 -----------------------------------------------------GCATCCG

LAINef*dd*-13 -----------------------------------------------------GCATCCG

LAINef*dd*-14 -----------------------------------------------------GCATCCG

LAI 541 TTAGAGTGGAGGTTTGACAGCCGCCTAGCATTTCATCACGTGGCCCGAGAGCTGCATCCG

*******

201 GluTryPheLysAsnCysEnd

LAINef*dd*-12 GAGTACTTCAAGAACTGCTGA

LAINef*dd*-13 GAGTACTTCAAGAACTGCTGA

LAINef*dd*-14 GAGTACTTCAAGAACTGCTGA

LAI 601 GAGTACTTCAAGAACTGCTGA

*********************

**Figure S3. Alignment of the *nef* coding region of plasma viral RNA.**

f
